# Effects of Center Metals in Porphines on Nanomechanical Gas Sensing

**DOI:** 10.3390/s18051640

**Published:** 2018-05-21

**Authors:** Huynh Thien Ngo, Kosuke Minami, Gaku Imamura, Kota Shiba, Genki Yoshikawa

**Affiliations:** 1 World Premier International (WPI) Research Center for Materials Nanoarchitectonics (MANA), National Institute for Materials Science (NIMS), 1-1 Namiki, Tsukuba 305-0044, Japan; MINAMI.Kosuke@nims.go.jp (K.M.); SHIBA.Kota@nims.go.jp (K.S.); YOSHIKAWA.Genki@nims.go.jp (G.Y.); 2 Materials Science and Engineering, Graduate School of Pure and Applied Science, University of Tsukuba, Tennodai 1-1-1 Tsukuba, Ibaraki 305-8571, Japan

**Keywords:** gas sensor, metalloporphine, MSS, olfactory, nanomechanical sensor

## Abstract

Porphyrin is one of the most promising materials for realizing a practical artificial olfactory sensor system. In this study, we focus on non-substituted porphyrins—porphines—as receptor materials of nanomechanical membrane-type surface stress sensors (MSS) to investigate the effect of center metals on gas sensing. By omitting the substituents on the tetrapyrrole macrocycle of porphyrin, the peripheral interference by substituents can be avoided. Zinc, nickel, and iron were chosen for the center metals as these metalloporphines show different properties compared to free-base porphine. The present study revealed that iron insertion enhanced sensitivity to various gases, while zinc and nickel insertion led to equivalent or less sensitivity than free-base porphine. Based on the experimental results, we discuss the role of center metals for gas uptake from the view point of molecular interaction. We also report the high robustness of the iron porphine to humidity, showing the high feasibility of porphine-based nanomechanical sensor devices for practical applications in ambient conditions.

## 1. Introduction

**Figure 1:**
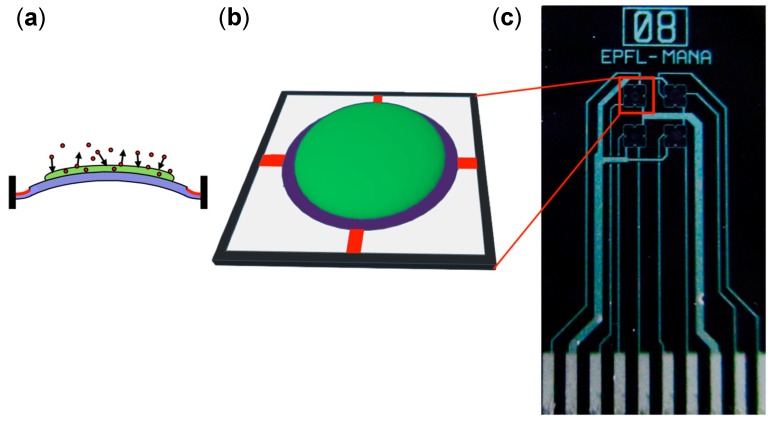
(**a**) Schematic illustration of the side view of the membrane. Blue, green, and red parts correspond to substrate, receptor layer, and embedded piezoresistors, respectively. (**b**) Schematic illustration of sensor channel. (**c**) Optical microscope image of a membrane-type surface stress sensor (MSS) chip.

Among sensors substituting the five senses, the sensors that can perceive physical stimuli, (e.g., light (eye), sound (ear), and pressure (skin)) have been extensively studied and developed. On the other hand, the sensors that detect chemical species, (e.g., tastes (tongue) and odors (nose)) still require intensive research because of a lack of comprehensive knowledge on the chemical interactions between the receptors and the analytes. Olfactory sensors, especially, have not been practically commercialized because of the complex perception mechanism of olfaction. Toward practical olfactory sensors, a sensing platform that meets various requirements such as high sensitivity, compactness, and low power consumption, is needed. For such a platform, we focus on nanomechanical membrane-type surface stress sensors (MSS) [1,2,3,4,5,6,7,8,9]. MSS are composed of thin silicon membranes suspended by four sensing bridges in which piezoresistors (Figure 1, red) are embedded. This structure allows highly sensitive electric read-outs of stress/strain induced by the sorption of gas molecules in the receptor layer (Figure 1). Since it has been confirmed that almost all solid materials exhibit mechanical deformation by gas sorption, MSS can detect diverse analytes by utilizing various kinds of materials as a receptor layer.

**Figure 2:**
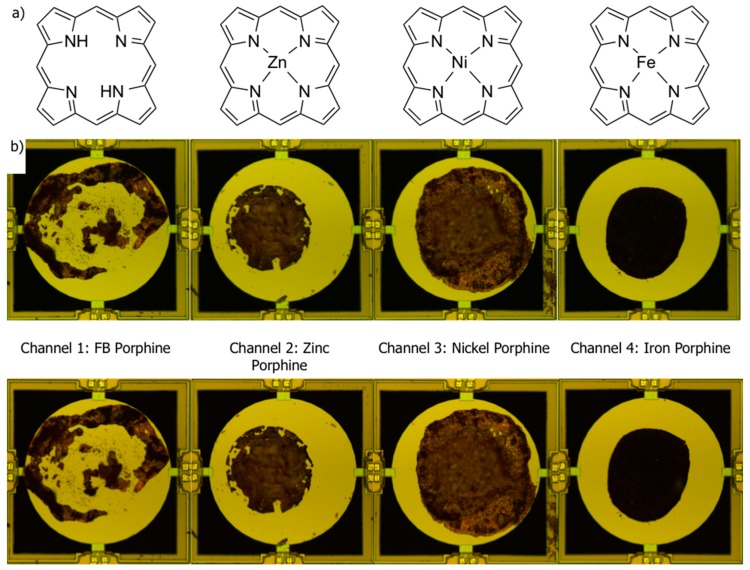
Porphine-based receptor materials coated on MSS channels. (**a**) Structures of free-base and metal coordinated porphines; (**b**) Optical microscope images of each MSS channel coated with porphines before (upper) and after (bottom) gas sensing measurements. Channel 1, free-base (FB) porphine; Channel 2, zinc porphine; Channel 3, nickel porphine; Channel 4, iron porphine.

As receptor materials for nanomechanical sensing, porphyrins are expected to be one of the most promising candidates. Porphyrins are the group of molecules containing tetrapyrrolic macrocycles, which possess a wide range of unique properties. For example, in biological systems, the iron porphyrin complex in heme plays a pivotal role in oxygen transport in mammals. This unique ability of porphyrins, especially metalloporphyrins, to bind specific guest molecules has attracted much attention in the field of chemical sensors because of their potential for enhancing selectivity [10,11]. Porphyrins and metalloporphyrins can bind to other molecules through different interactions including van der Waals forces, hydrogen bonding, π−π interactions, and coordination to central metal ions [12]. Although the dominant interaction depends on each condition, these interactions with other molecules induce significant changes in mass, density, work functions, and optical properties. Thus, porphyrins can be utilized in various kinds of sensing systems [13,14,15]. Di Natale and Paolesse [16,17,18,19] have explored the potential of porphyrins for gas sensing by employing quartz crystal microbalance (QCM), electrical signaling, and potentiometric sensor arrays. Rao et al. [20] reported the piezoresistive cantilever coated with Fe(III)-porphyrin for carbon monoxide sensing. Recently, we have also reported a free-base porphyrin covalently bonded to a highly networked silica structure, so-called silica flake-shell capsules for acetone sensing using MSS [21], and investigated the dependence of structural difference of silicas on sensing performance. Table S1 in Supplementary Materials summarizes the types of porphyrins and sensing techniques of these previous studies. Although these previous studies have shown the high potential of porphyrins for gas sensing, fundamental aspects of gas uptake in porphyrins still need to be pursued. The effects of different center metals in metalloporphyrins have been intensively studied with substituted porphyrins [16,17,18,22,23,24,25]. Although the comparison with different metals in the same porphyrin system can show the inherent effect of each metal, the signal could be enhanced or reduced due to the substituents. The peripheral substituents can interact with guest molecules through several interactions such as steric effects, π-π interactions, and hydrogen bonding. Moreover, the substituents alter the electronic properties of the metal-porphyrin complex, leading to a different binding affinity of axial ligands to metal centers. To understand the fundamental mechanism of porphyrin-based gas sensing and develop an advanced sensing platform, it is necessary to clarify the essential role of center metals by eliminating the effects of peripheral substituents. In this study, we investigated the sensing properties of porphine and metalloporphines as receptor materials of nanomechanical sensors. Porphine is the central structure of porphyrins—a porphyrin without substituents. By using porphine and metalloporphines, we can exclude the effects of peripheral substituents and focus on the sole effect of the center metal ions and the central polypyrrole macrocycle on gas uptake. We measured gas sensing properties of four porphine derivatives: free-base (FB) porphine, nickel porphine, zinc porphine, and iron porphine (Figure 2a) [26]. According to the naming rules of the International Union of Pure and Applied Chemistry (IUPAC), the names of free-base (FB) porphine, nickel porphine, zinc porphine, and iron porphine are 21,22,23,24-tetraazapentacyclo[16.2.1.1^3,6^.1^8,11^.1^13,16^]tetracosa-1,3,5,7,9,11(23),12,14,16,18(21),19-undecaene, (*SP*-4-1)-[21*H*,23*H*-Porphinato(2-)-κN^21^, κN^22^, κN^23^, κN^24^]nickel, (*SP*-4-1)-[21*H*,23*H*-Porphinato(2-)-κN^21^, κN^22^, κN^23^, κN^24^]zinc, (*SP*-4-1)-[21*H*,23*H*-Porphinato(2-)-κN^21^, κN^22^, κN^23^, κN^24^]iron, respectively. The IUPAC names of the porphines are summarized in Table S2 in Supplementary Materials. The results showed that metal insertion leads to significant changes in sensitivity, reflecting the different coordination preference of center metal ions to axial guest molecules in the porphine complex. Moreover, we also demonstrated humidity resistances up to 90% relative humidity, showing the possibility for practical application of porphine-based nanomechanical sensors in ambient conditions.

## 2. Materials and Methods

All chemicals were purchased from Tokyo Chemical Industry Co. Ltd., Sigma–Aldrich Co., Wako Pure Chemical Industries, Ltd., Kanto Chemical Co. Ltd., and Nacalai Tesque, Inc. The porphines were synthesized with reagent grade or higher grade chemicals, which were used as received. For gas sensing measurements, pure water, ethanol, 1-hexanol, hexanal, *n*-heptane, methylcyclohexane, toluene, ethyl acetate, acetone, chloroform, aniline, and propionic acid were purchased as analytical grade chemicals and used as received. As the center metal ions, nickel, zinc, and iron were selected owing to their distinctive coordination [27] and hydration properties [28]. Free-base porphine and nickel-, zinc-, and iron-metalloporphines were synthesized according to the literature [29,30,31,32,33]. The porphines were characterized by ^1^H Nuclear Magnetic Resonance (NMR) measured with a JEOL AL 300 FT-NMR spectrometer using tetramethylsilane (TMS) as an internal reference.

In this study, we used a 4-channel MSS chip as a chemical sensor. Each channel was coated with the different porphine derivative by inkjet spotting. Before coating, the MSS chip was washed with water, acetone, and isopropyl alcohol. The porphines were dissolved in *N*,*N*′-dimethylformamide (DMF) at a concentration of 1 mg/mL. Each solution of porphines was deposited on each channel of the MSS chip with an inkjet spotter (LaboJet-500SP) equipped with a nozzle (IJHBS-300). Both the inkjet spotter and the nozzle were purchased from MICROJET Corporation. The same parameters (see Table S3 in Supplementary Materials) were applied for every solution to ensure uniform deposition on each channel (Figure 2b). The MSS chip coated with the four porphines was mounted in a Teflon chamber, and the chamber was carefully sealed with O-rings. The chamber was placed in a constant temperature bath kept at 25 °C. The sample gases—the vapors of the 12 solvents generated via bubbling—were injected in to the chamber with a gas flow system equipped with three mass flow controllers (MFCs). Nitrogen was used as a carrier gas. The concentrations of the sample gases were calibrated by measuring the decrease in the weight of the solvents before and after a gas flow. The relative humidity (RH) was controlled by providing a saturated water vapor to the gas flow line. The concentration of the sample gases and the humidity of the carrier gas were adjusted by controlling the flow rates of the three MFCs. The total gas flow rate of the three MFCs was set at 100 standard cubic centimeters per minute (sccm). The surface stress caused by the gas sorption/desorption in the receptor layer was electrically read by a Wheatstone bridge circuit consisting of the piezoresistors embedded on the bridges [1]. In the present study, a voltage of −0.5 V was applied to the circuit, and the relative resistance changes of piezoresistors were detected as output signals.

The gas measurements were performed through 10 cycles of 10-s sample gas injection and 10-s nitrogen purge. The sample gases were diluted to 2% or 10% of their saturated vapor concentration. The carrier gas was humidified at 0%, 10%, 40%, 70% and 90% RH.

## 3. Results and Discussion

As a nanomechanical sensor detects stress or deformation caused by expansion and shrinkage of a receptor layer, a sensing signal depends on morphology of the receptor layer. However, the previous study showed that the sensing signals of an MSS are less affected by the roughness of the receptor layer than those of a cantilever-type nanomechanical sensor, as long as the volume of the receptor layers was constant [5]. Therefore, the difference in coating morphology of the porphines (Figure 2b) has limited effects on the sensing response because the volume of each porphine on a membrane was strictly controlled during the inkjet-spotting. Figure 2b shows the optical microscope images of the four channels before (upper) and after (bottom) the measurements, respectively. It was clearly observed that the receptor layers did not deteriorate through the measurements, showing the high robustness to the chemicals.

**Figure 3:**
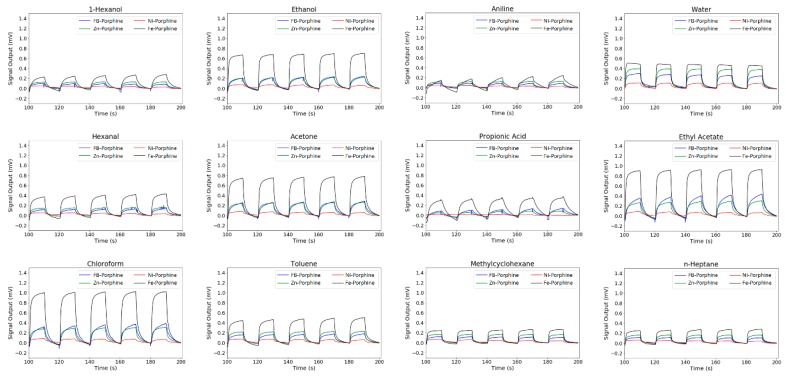
Sensing signals of porphines to gases. The last five cycles of sample gas injection and nitrogen gas purge are shown. Temperature, concentration of gases and relative humidity were 25 °C, 10% in partial vapor concentration and 0% RH, respectively.

Figure 3 shows the typical sensing signals obtained through the measurements (10% in partial vapor concentration, 0% RH, 25 °C). Only the last five cycles were used for analysis because the first few cycles tended to be disturbed by the history of the previous measurement. The baseline was subtracted for every signal so that the signal output at 180 s becomes 0 mV. For free-base porphine, two main features can be seen from Figure 3. Firstly, free-base porphine was highly sensitive to most of the oxygen-containing molecules. Secondly, free-base porphine showed the highest sensitivity to toluene among the hydrocarbons. These two features can be attributed to the two different interactions between the porphines and gas molecules. One was the hydrogen bonding with the central NHs, which contributes to the high sensitivity to oxygen-containing molecules. The other is the aromatic interaction with the peripheral pyrroles of porphines, resulting in the high affinity to toluene.

**Figure 4:**
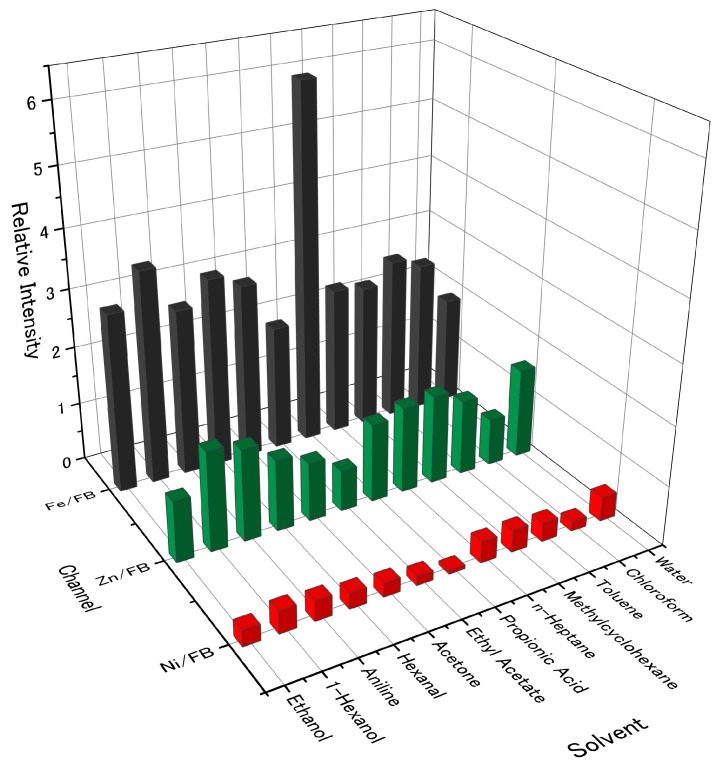
Relative intensity of metalloporphines compared to free-base porphine.

The sensing properties of porphine were significantly changed by metal insertion. For almost all the measured gases, the sensitivity of the metalloporphines followed a general trend: nickel < free-base ~ zinc < iron porphines. Figure 4 shows the relative intensity of each metalloporphine to free-base porphine. The signal intensities of the four porphines to the sample gases are shown in the Supplementary Materials (Figure S4). By replacing the central hydrogen atoms with a metal ion, the dominant interaction between a gas species and a metalloporphine becomes the coordination interaction with the central metal ion. To the hydrocarbons, which have no lone pairs or hydrogen bonding sites, the metalloporphines showed similar selectivity as free-base porphine. Toluene showed the highest intensity because of the aromatic interaction between its benzene ring and the peripheral pyrroles of a porphine. Among the three metals, nickel insertion drastically reduced the signal intensity. In general, most of oxygen-containing molecules could bind to the center metal ion in nickel porphyrins with substituents, as the substituents could act as electron withdrawing moieties, which enhanced binding affinity to the axial coordination of nickel [34]. In contrast, the nickel center in porphine did not show strong coordination with gas molecules because a porphine molecule has no substituents; the lack of withdrawing moieties led to a change in the coordination structure from a pyramidal geometry to a square-planar geometry, which showed no axial coordination.

Zinc porphine showed similar signal intensity to free-base porphine (Figure 4). However, it can be seen from Figure 4 that the intensity ratio slightly deviated from 1.0, reflecting the altered porphine-gas interaction by zinc insertion. Zinc porphine generally had a strong pentadentate coordination mode, which allowed one axial ligand to be complexed to the central zinc atom [35]. When two ligands were coordinated to the zinc center, zinc porphine showed a six-coordinated structure with two axial ligands, in which the binding energy of a second complexation was much lower than that of the first [36]. Thus, it was more likely that zinc porphine took up only one axial ligand when the concentration of a sample gas was low. Compared to free-base porphine, zinc porphine exhibited slightly different intensity owing to its different electronic structure. Alcohols and amines have lone pairs, which favor axial coordination with the zinc porphine. This effect prevails over hydrogen bonding with free-base porphine, leading to the higher intensity of the zinc-porphine for the alcohols and aniline than free-base porphine [37,38,39,40,41].

**Figure 5:**
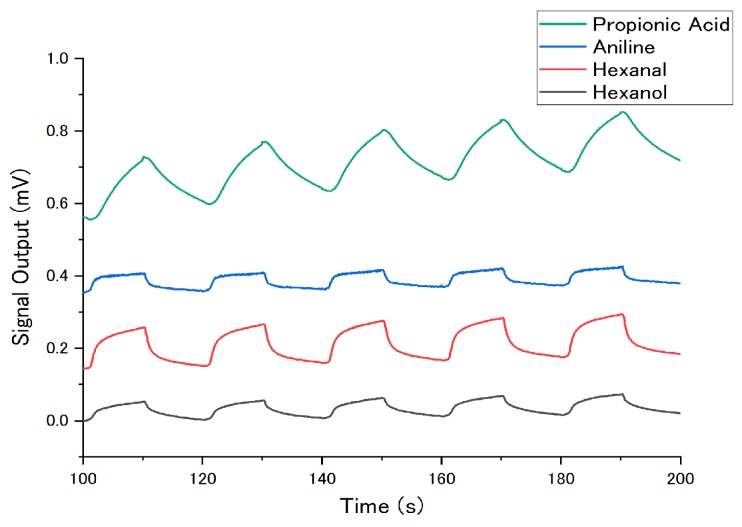
Sensing signals of iron porphine at 25 °C, 2% in partial vapor concentration, 0% RH.

Among the three metalloporphines, iron porphine showed the highest intensity to all the measured gases, especially to propionic acid (Figure 4). This was expected as the central iron atom can bind to two extra axial ligands to form octahedral hexadentate coordination [42]. This was in agreement with the literature reporting higher ligand binding of iron porphyrin than that of zinc and nickel derivatives [43]. Moreover, the iron porphine could detect all of the measured gases at a low concentration (2% in partial vapor concentration) at 0% RH. In particular, for hexanal, propionic acid, aniline, and 1-hexanol, iron porphine could detect these gases at a concentration of 259, 19, 8 and 9 ppm, respectively (Figure 5), because of the strong coordination with two axial ligands to the iron center. Note that iron porphine also exhibited enhanced intensity for gas species with which the center iron atom could not form coordination bonds: n-heptane, methylcyclohexane, and toluene. This is because the electronic structure of the central polypyrrole macrocycle was also changed by iron insertion. Owing to such changes in the electronic structure, interactions between central polypyrrole macrocycle and gases can be also affected, leading to the enhanced intensity to the gas species for which the CH/π interaction and the π-π interaction were dominant.

**Figure 6:**
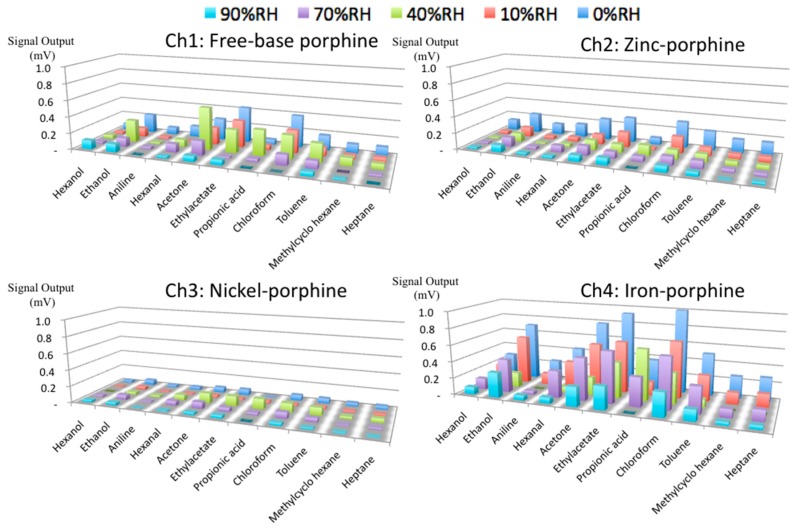
Sensitivity at 25 °C and 10% in partial vapor pressure at different humidity.

**Figure 7:**
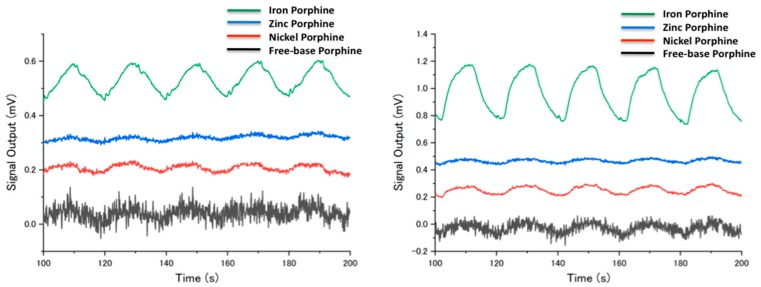
Sensing signals to 1-hexanol (**left**) and propionic acid (**right**) sensing at 25 °C, 10% in partial vapor concentration, 70% RH. The last five cycles of sample gas injection and nitrogen gas purge are shown.

**Figure 8:**
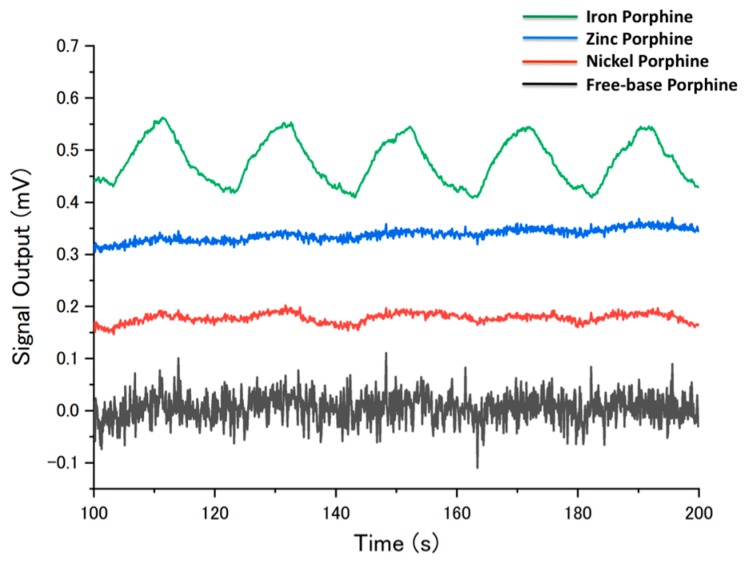
Sensing signals to propionic acid at 25 °C, 2% in partial vapor pressure, 70% RH. The last five cycles of sample gas injection and nitrogen gas purge are shown.

The different coordination behavior of the center metals in porphines also affected their stability against humidity (Figure 6). The sensing signals of the four porphines to the sample gases (10% in partial concentration) measured at different humidity is shown in the Supplementary Materials (Figures S5–S8). In a humidified condition, the sensing signals were generally suppressed because water molecules compete with analytes at the coordination site of porphines. However, toluene still showed relatively high signal intensity even at high humidity because the π-π interaction, that is a dominant interaction between toluene and porphines, was not interfered by the presence of water. The low sensitivity of nickel porphine was further suppressed by humidity because of its square-planar coordination mode with no vacant axial coordination site on the nickel center. For free-base porphine at 10% in partial vapor concentration, alcohols, acetone, ethyl acetate, and chloroform could still be detected, indicating the formation of hydrogen bonds competing with water. Zinc porphine also lost its intensity at high humidity, while the gases with electron donor moieties were still detected because of the strong axial coordination to the zinc center. Importantly, iron porphine maintained its sensitivity for most of the measured gases, owing to its two strong axial coordination sites. These different behaviors between zinc and iron porphines can be supported by two separate studies by Schalk [35] and Kang [42]. They calculated the binding energies of water molecules to zinc and iron porphyrinic systems and showed that zinc porphine has a higher affinity to water molecules than that of iron porphine. Therefore, water molecules axially coordinated to the iron center can be easily replaced by gas molecules like the oxygen-transport function of hemoglobin in a physiological environment. Particularly for hexanol and propionic acid, the iron porphine retains the sensitivity up to 70% RH (Figure 7). Even at 2% in partial vapor concentration (19 ppm), propionic acid could be clearly detected by iron porphine (Figure 8).

## 4. Conclusions

In conclusion, we have studied the properties of porphines and metalloporphines as receptor materials of nanomechanical sensors. We investigated the effect of metal centers in porphines on the sensing ability to various gases by using a nanomechanical MSS. By using porphines, we avoid any interfering effects of the peripheral substituents. We have showed that nickel and zinc insertions into porphines gave rise to equal to or less sensitivity than free-base porphine. In contrast, we revealed that iron porphine exhibited enhanced sensitivity owing to its high capability of ligand binding and modified electronic structure according to the iron center. Moreover, we also demonstrated the high humidity-resistance sensing capability of iron porphine. Even in a humidified condition (70% RH), propionic acid can be clearly detected with iron porphine at as low as 19 ppm. The present study can be used as a reference for future investigations where the influence of metals in porphyrinoids plays an important role, leading to advanced sensing applications including receptor materials for an artificial olfactory sensor system.

## Supplementary Materials

The supplementary materials are available online at http://www.mdpi.com/1424-8220/18/5/1640/s1.
