# Supplementary material for: Effects of Center Metals in Porphines on Nanomechanical Gas Sensing
Source: Sensors (Basel). 2018 May 21;18(5):1640. doi: 10.3390/s18051640 (PMC5982686; doi:10.3390/s18051640)
Supplement: Supplementary file 1 [file sensors-18-01640-s001.pdf]

Supplementary Materials

Effects of Center Metals in Porphines on Nanomechanical Gas Sensing

Huynh Thien Ngo[1*], Kosuke Minami[1], Gaku Imamura[1*], Kota Shiba[1] and Genki Yoshikawa[1,2]

[1] World Premier International (WPI) Research Center for Materials Nanoarchitectonics (MANA), National Institute for Materials Science (NIMS), 1-1 Namiki, Tsukuba 305-0044, Japan

[2] Materials Science and Engineering, Graduate School of Pure and Applied Science, University of Tsukuba, Tennodai 1-1-1 Tsukuba, Ibaraki 305-8571, Japan

* Correspondence: NGO.Huynhthien@nims.go.jp (H.T.N.), Tel.: +81- 29-851-3354; IMAMURA.Gaku@nims.go.jp (G.I.), Tel.: +81-29-860-4988

Previous studies on porphyrin-based chemical sensors

Previous studies using porphyrin as sensing materials are summarized in Table S1. Substituents (the substituent positions are indicated in Figure S1), type of sensors, and references are listed.

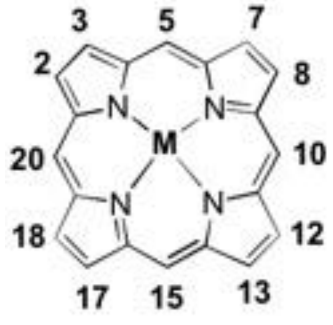

Figure S1 Molecular structure of porphine (non-substituted porphyrin) with carbon number.

Table S1. Summary of the previous researches.

Substituents	Center Metals	Type of Sensors	References
5,10,15,20-(4-heptyloxyphenol)	HH	QCM	[S1]
2,18-diethyl-3,7,8,12,13,17-hexamethyl-10-pentafluorophenyl	Cu	QCM	[S1]
5,10,15,20-(4-heptyloxyphenol)	Co	QCM	[S1]
5,10,15,20-(4-heptyloxyphenol)	Mn	QCM	[S1]
5,10,15,20-tetrakis[3,4-bis (2-ethylhexyloxy)phenyl]	HH	Optical spectrometer	[S2]
5,10,15,20-tetrakis[3,4-bis (2-ethylhexyloxy)phenyl]	Au, Co, Mn, Mg, Sn	Optical spectrometer	[S3]
5,10,15,20-tetraphenyl	HH	Optical spectrometer	[S4]
5,10,15,20-tetraphenyl Functionalized with single walled carbon nanotube	Fe	Conductivity measurement	[S5]
2,3,7,8,12,13,17,18-octaethyl Langmuir-Blodgett film	Ru	Conductivity measurement	[S6]
5,10,15,20-tetraphenyl	Rh	Conductivity measurement	[S7]
5,10,15,20-tetraphenyl	Ru	Conductivity measurement	[S7]
5,10,15,20-tetraphenyl	Mn	Conductivity measurement	[S7]
5,10,15,20-tetraphenyl	Co	Conductivity measurement	[S7]
5,10,15,20-tetraphenyl	DSn	Conductivity measurement	[S7]
5,10,15,20-tetra-(4-NO_2-phenyl)	Co	Conductivity measurement	[S7]

5,10,15,20-tetra-(4-MeOOC-phenyl)	Co	Conductivity measurement	[S7]
5,10,15,20-tetra-(4-butyloxyphenyl)	Zn	QCM	[S8]
5,10,15,20-tetraphenyl	HH, Zn, Sn	Optical spectrometer	[S9]
5,10,15,20-tetra-(4-MeOOCphenyl)	HH	Optical spectrometer	[S9]
5-(4-aminophenyl)-10,15,20-triphenyl	HH, Zn	Optical spectrometer	[S9]
5,10,15,20-tetra-(4-hydroxyphenyl)	HH	Optical spectrometer	[S10]
2,3,7,8,12,13,17,18-octaethyl Diyne-bridged dimer	Ni	Conductivity measurement	[S11]
5,10,15,20-tetraphenyl	Not specified	Optical spectrometer	[S12]
5,10,15,20-tetraphenyl	Not specified	Optical spectrometer	[S13]
5,10,15,20-tetraphenyl	Sn, Co, Cr, Mn, Fe, Cu, Ru, Zn, Ag, HH	Optical spectrometer	[S14]
5,10,15,20-tetra [3,4-(dimethyloxy)phenyl]	Fe	Piezoresistive SU-8 cantilever	[S15]
5-[4-(N-(3-Triethoxysilylpropyl benzamido))]-10,15,20-triphenyl	HH, Co, Zn, Cu, Ni	MSS	[S16]

Nomenclature of Porphines

Table S2. IUPAC names of the porphines

Porphines	IUPAC Names
Porphine	21,22,23,24-tetraazapentacyclo[16.2.1.1^{3,6}.1^{8,11}.1^{13,16}]tetracosa-1,3,5,7,9,11(23),12,14,16,18(21),19-undecaene
Nickel Porphine	(*SP*-4-1)-[21*H*,23*H*-Porphinato(2-)-κN^{21}, κN^{22}, κN^{23}, κN^{24}]nickel
Zinc Porphine	(*SP*-4-1)-[21*H*,23*H*-Porphinato(2-)-κN^{21}, κN^{22}, κN^{23}, κN^{24}]zinc
Iron Porphine	(*SP*-4-1)-[21*H*,23*H*-Porphinato(2-)-κN^{21}, κN^{22}, κN^{23}, κN^{24}]iron

Synthesis of the Porphines

Firstly, 5,10,15,20-tetrabutylporphyrin was synthesized [S17]. Then, dealkylation of 5,10,15,20-tetrabutylporphyrin by concentrated sulfuric acid provided non-substituted porphine [S18]. The metallation of non-substituted porphine followed the literature; nickel porphine [S19], iron porphine [S20] and zinc porphine were synthesized by a different procedure [S21].

The NMR spectra were measured in deuterated dimethylformamide for a better solubility (Figure S2). Unfortunately, iron porphine does not dissolve well in any of the available deuterated solvents. The signal for the inner NH-protons of freebase porphine at -3.938 ppm disappears after metallation. Metal insertion caused the proton shift to the higher field. The meso-protons are shifted from 10.699 ppm (freebase porphine) to 10.499 ppm (zinc porphine) and 10.465 ppm (nickel porphine). The beta-protons were also shifted from 9.806 ppm (freebase porphine) to 9.702 ppm (zinc porphine) and 9.622 ppm (nickel porphine). When the spectrum of iron porphine is enlarged and zoomed in at the aromatic region (left upper corner), a small peak at 8.783 ppm is observed. Due to the low solubility we cannot assign with certainty this peak as meso- or beta-protons.

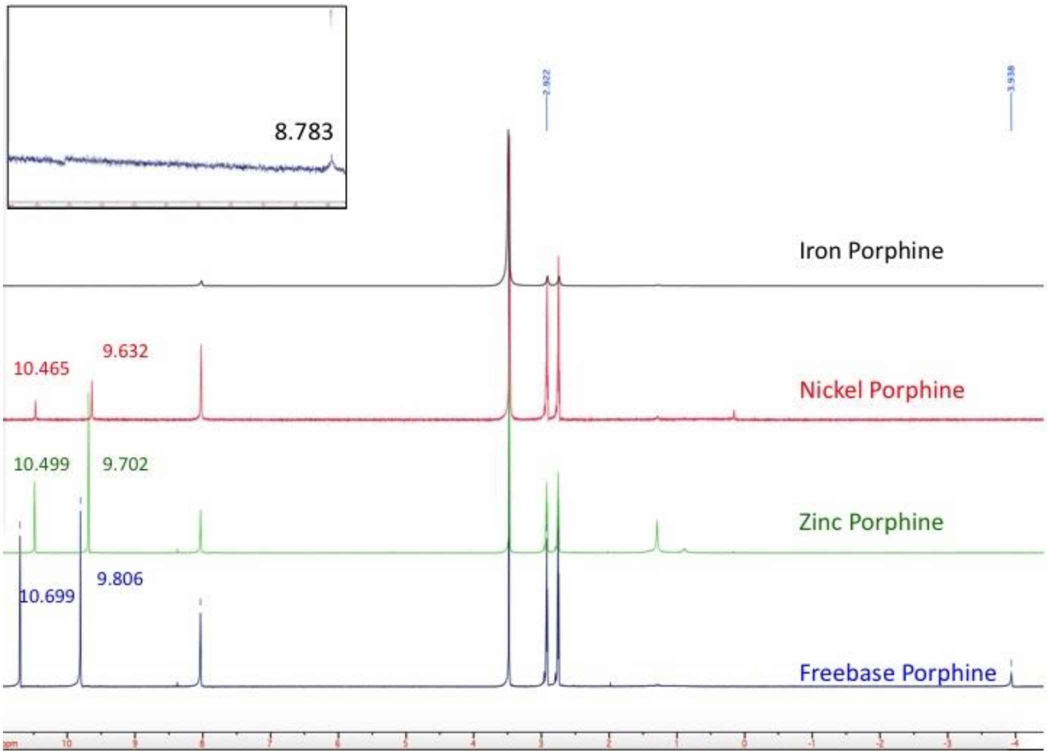

Figure S2: ^{1}H NMR (DMF-D$_7$, 20°C) of free-base porphine (blue), zinc porphine (green), nickel porphine (red) and iron porphine (black).

The mass spectrum (MALDI-TOF) of iron porphine (Figure S3) gives the right molecular mass:

Calculated [M+H$^+$] = 365.19

Found [M+H$^+$] = 365.06

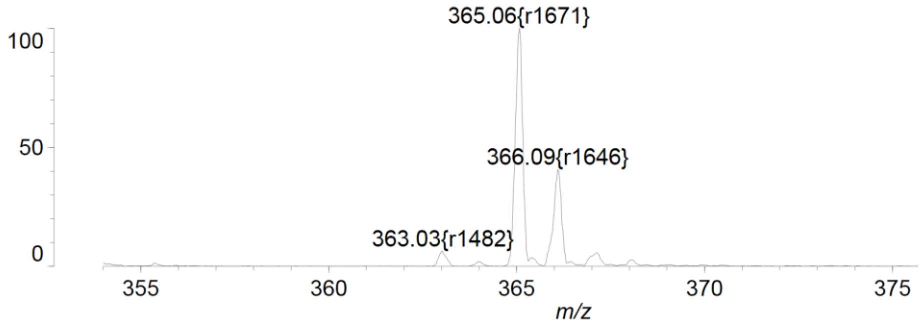

Figure S3: Mass spectrum of iron porphine.

Optimized Parameters for Inkjet Spotting

Table S3. Parameters for inkjet spotting for coating receptor layer.

Parameter	Value
Injection speed	~5 m/s
Volume of a droplet	~300 pL
Stage temperature	80 °C

Signal Intensity at 0% RH

Signal intensities of the four porphines to the 12 sample gases were summarized in Figure S4. The concentration of the sample gases was set at 10% in their partial concentration, and the relative humidity was set at 0% RH.

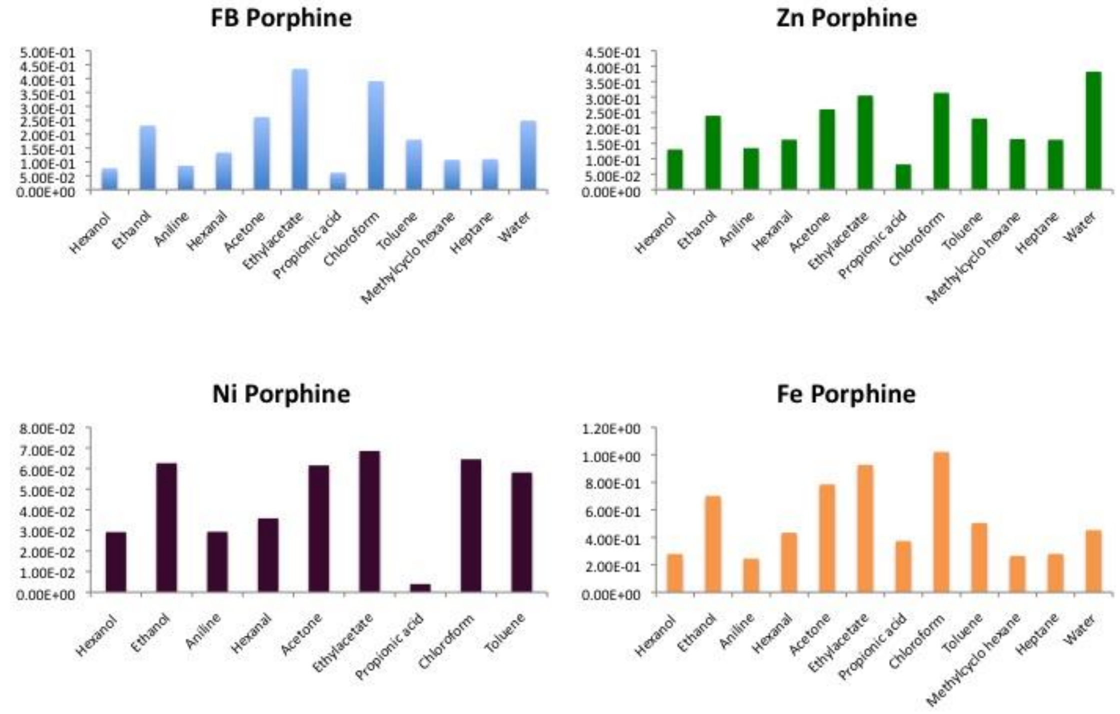

Figure S4. Intensity of four porphines to sample gases at 25 °C, 10% in partial vapor pressure, 0%RH.

Raw Sensing Signals

Figures S5-S8 show the sensing signals of the four porphines to the 12 sample vapors measured at different humidity: 0, 10, 40, 70, and 90%RH. The concentration of the sample gases was fix at 10% of their partial vapor concentration. The last five cycles of the sample gas injection and the nitrogen purge are shown in Figures S1-S5. The baseline is not subtracted for each measurement.

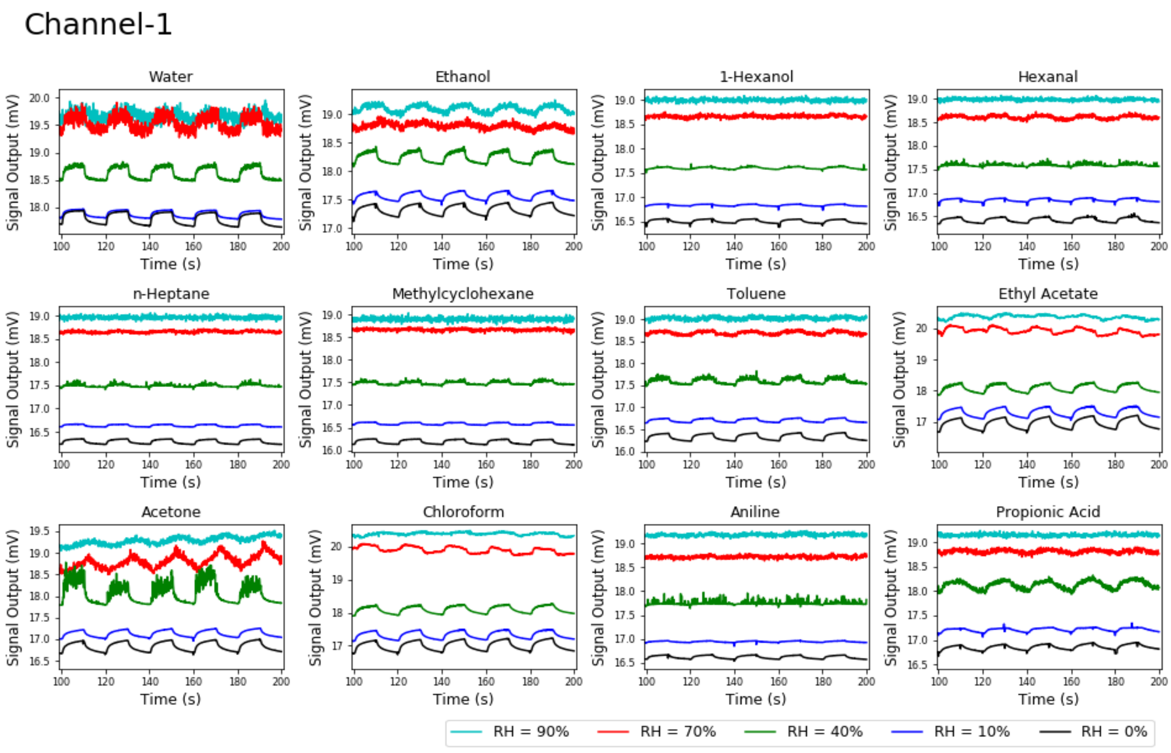

Figure S5. Sensing signals of free-base porphine to samples vapors at different humidity. The concentration of sample gases was fix at 10% in partial vapor concentration.

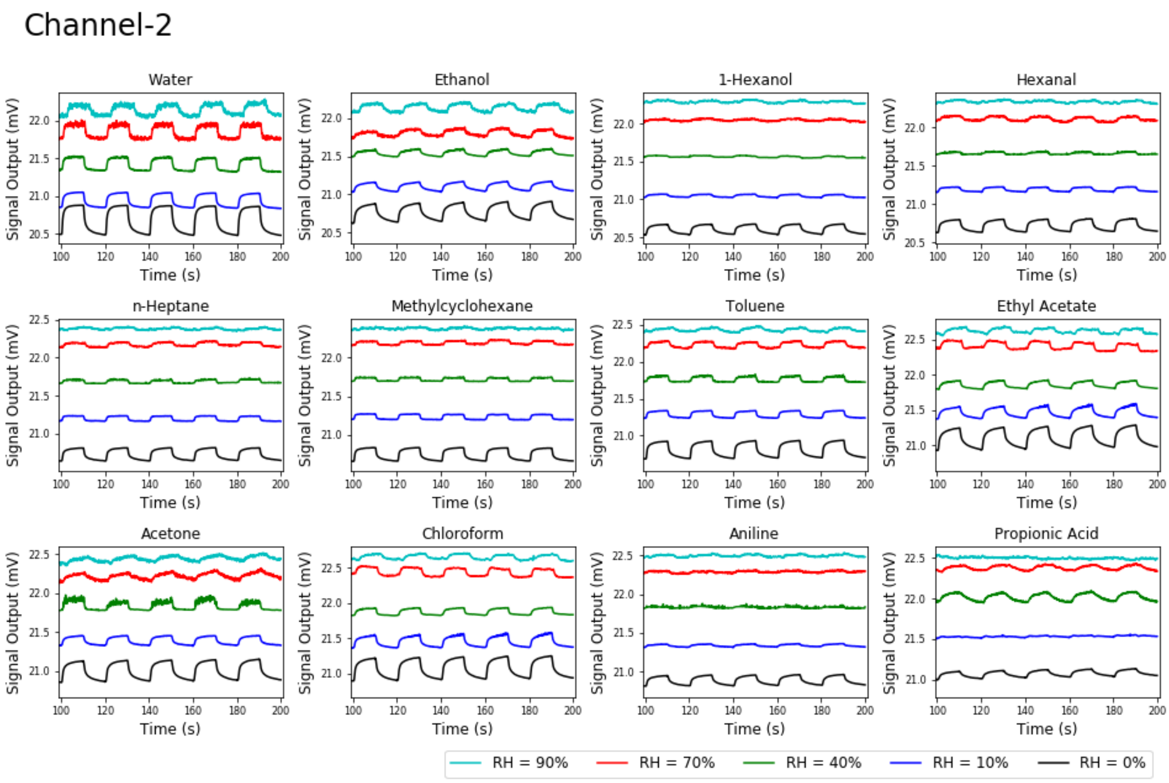

Figure S6. Sensing signals of zinc porphine to samples vapors at different humidity. The concentration of sample gases was fix at 10% in partial vapor concentration.

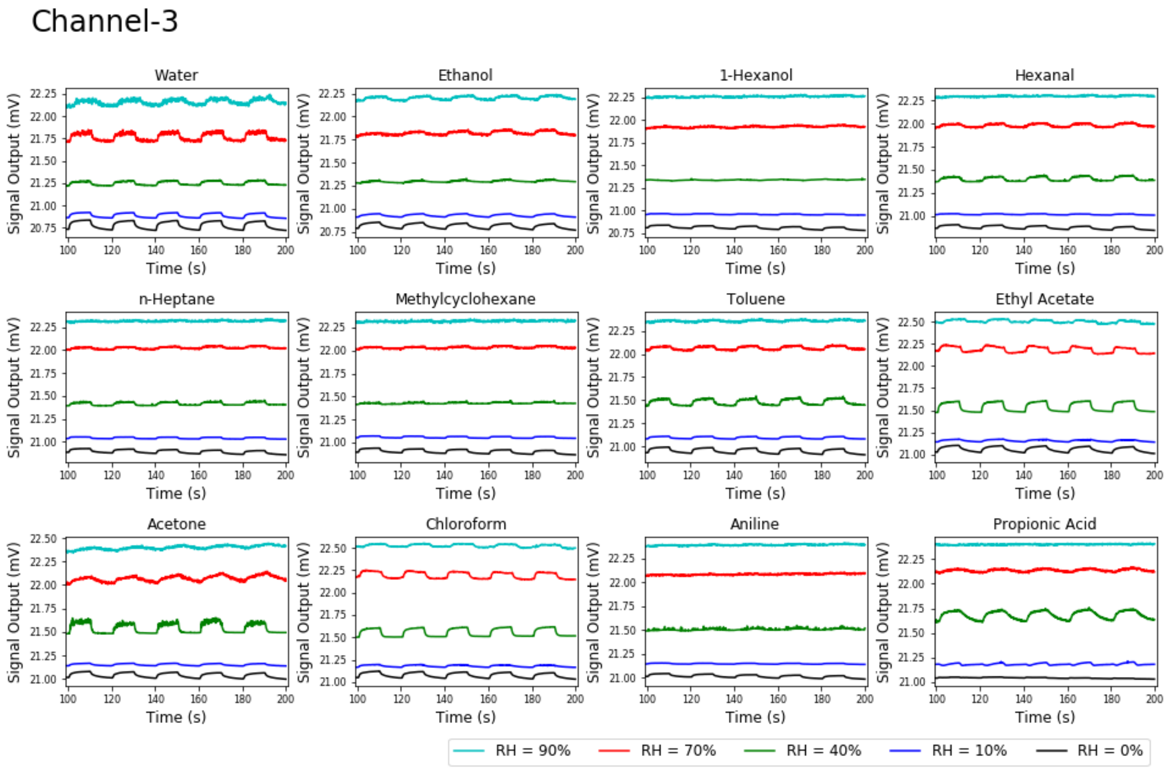

Figure S7. Sensing signals of nickel porphine to samples vapors at different humidity. The concentration of sample gases was fix at 10% in partial vapor concentration.

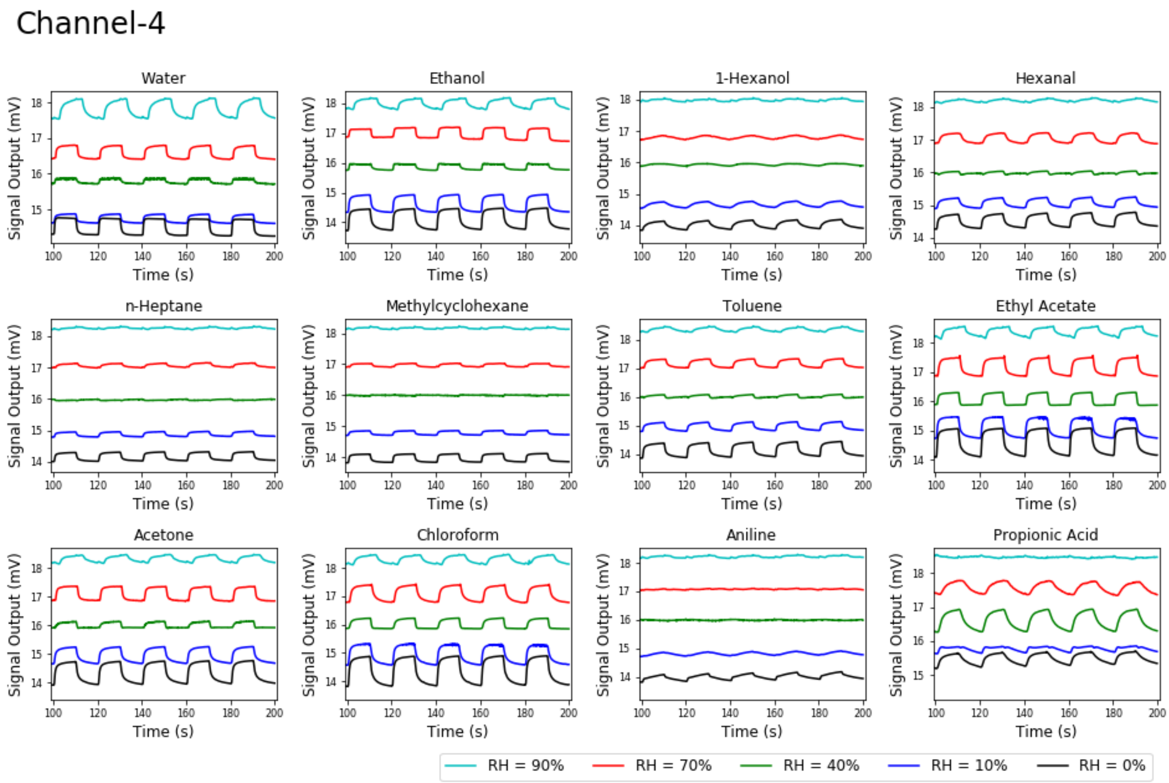

Figure S8. Sensing signals of iron porphine to samples vapors at different humidity. The concentration of sample gases was fix at 10% in partial vapor concentration.

References

[S1] Di Natale, C.; Paolesse, R.; Macagnano, A.; Mantini, A.; Goletti, C.; D'Amico, A., Characterization and design of porphyrins-based broad selectivity chemical sensors for electronic nose applications.

Sens. and Actuators B: Chemical **1998**, *52*, 162–168.

[S2] Evyapan, M.; Dunbar, A.D.F. "Controlling surface adsorption to enhance the selectivity of porphyrin based gas sensors"

Applied Surface Science **2016**, *362*, 191–201.

[S3] Dunbar, A. D. F.; Brittle, S.; Richardson, T. H.; Hutchinson, J.; Hunter, C. A., "Detection of Volatile Organic Compounds Using Porphyrin Derivatives"

J. Phys. Chem. B **2010**, *114*, 11697–11702.

[S4] Kladsomboon, S.; Kerdcharoen, T. "A method for the detection of alcohol vapours based on optical sensing of magnesium 5,10,15,20-tetraphenyl porphyrin thin film by an optical spectrometer and principal component analysis"

Analytica Chimica Acta **2012,** *757,* 75–82

[S5] Rushi, A.; Datta, K.; Ghosh, P.; Mulchandani, A.; Shirsat, M.D. "Iron tetraphenyl porphyrin functionalized single wall carbon nanotubes for the detection of benzene"

Materials Letters **2013**, *96*, 38–41.

[S6] Zhu, D.-G.; Cui, D.-F.; Petty, M. C. "Gas sensing using Langmuir-Blodgett films of a ruthenium porphyrin"

Sensors and Actuators B, **1993**, *12*, 111–114.

[S7] Di Natale, C.; Macagnano, A.; Repole, G.; Saggio, G.; D'Amico, A.; Paolesse, R.; Boschi, T., "The exploitation of metalloporphyrins as chemically interactive material in chemical sensors. *Mater. Sci. Eng.: C* **1998**, *5*, 209–215.

[S8] Macagnano, A.; Zampetti, E.; Pistillo, B. R.; Pantalei, S.; Sgreccia, E.; Paolesse, R.; d'Agostino, R., Double layer sensors mimic olfactive perception: A case study. *Thin Solid Films* **2008,** *516*, 7857– 7865.

[S9] Xu, H.; Cao, K.-D.; Ding, H.-B.; Zhong, Q.-F.; Gu, H.-C.; Xie, Z.-Y.; Zhao, Y.-J.; Gu, Z.-Z., Spherical Porphyrin Sensor Array Based on Encoded Colloidal Crystal Beads for VOC Vapor Detection. *ACS Applied Materials & Interfaces* **2012,** *4* (12), 6752– 6757

[S10] Paske, A. C.; Earl, L. D.; O'Donnell, J. L., "Interfacially polymerized metalloporphyrin thin films for colorimetric sensing of organic vapors." *Sensors and Actuators B: Chemical* **2011**, *155*, 687– 691.

[S11] Arnold, D. P.; Manno, D.; Micocci, G.; Serra, A.; Tepore, A.; Valli, L., "Gas-sensing properties of porphyrin dimer Langmuir–Blodgett films." *Thin Solid Films* **1998,** *327-329*, 341–344.

[S12] Janzen, M. C.; Ponder, J. B.; Bailey, D. P.; Ingison, C. K.; Suslick, K. S., "Colorimetric Sensor Arrays for Volatile Organic Compounds" *Anal. Chem.* **2006**, *78*, 3591–3600.

[S13] Lim, S. H.; Kemling, J. W.; Feng, L.; Suslick, K. S., "A colorimetric sensor array of porous pigments." *Analyst* **2009,** *134* (12), 2453–2457.

[S14] Rakow, N. A.; Suslick, K. S., A colorimetric sensor array for odour visualization. *Nature* **2000,** *406*, 710–713.

[S15] Reddy, C. V. B.; Khaderbad, M. A.; Gandhi, S.; Kandpal, M.; Patil, S.; Chetty, K. N.; Rajulu, K. G.; Chary, P. C. K.; Ravikanth, M.; Rao, V. R., Piezoresistive SU-8 Cantilever With Fe(III)Porphyrin Coating for CO Sensing. *IEEE Trans. Nanotechnol.* **2012,** *117*, 701-706.

[S16] Osica, I.; Imamura, G.; Shiba, K.; Ji, Q.; Shrestha, L. K.; Hill, J. P.; Kurzydłowski, K. J.; Yoshikawa, G.; Ariga, K., Highly Networked Capsular Silica–Porphyrin Hybrid Nanostructures as Efficient Materials for Acetone Vapor Sensing. *ACS Appl. Mater. Interfaces.* **2017,** *9*, 9945-9954.

[S17] Senge Mathias, O.; Bischoff, I.; Nelson Nora, Y.; Smith Kevin, M., Synthesis, reactivity and structural chemistry of 5,10,15,20-tetraalkylporphyrins. *J. Porphyrins.Phthalocyanines* **1999,** *3*, 99-116.

[S18] Senge Mathias, O.; Bischoff, I.; Nelson Nora, Y.; Smith Kevin, M., Synthesis, reactivity and structural chemistry of 5,10,15,20-tetraalkylporphyrins. *J. Porphyrins.Phthalocyanines* **1999,** *3*, 99-116.

[S19] Neya, S.; Funasaki, N., meso-Tetra(tert-butyl)porphyrin as a precursor of porphine. *Tetrahedron Lett.* **2002,** *43*, 1057-1058.

[S20] Liu, P. P.; Feng, Y. Q.; Gu, C. Z.; Meng, S. X.; Zhang, B., The facile synthesis of 5-formylporphyrin. *Chin. Chem. Lett.* **2012,** *23*, 505-508.

[S21] Neya, S.; Funasaki, N.; Sato, T.; Igarashi, N.; Tanaka, N., Structural Analysis of the Myoglobin Reconstituted with Iron Porphin. *J. Biol. Chem.* **1993,** *269*, 7.

[S22] Dogutan, D. K.; Ptaszek, M.; Lindsey, J. S., Direct Synthesis of Magnesium Porphine via 1-Formyldipyrromethane. *J. Org. Chem.* **2007,** *72*, 5008-5011.
